# Lsr operon is associated with AI-2 transfer and pathogenicity in avian pathogenic *Escherichia coli*

**DOI:** 10.1186/s13567-019-0725-0

**Published:** 2019-12-12

**Authors:** Jiakun Zuo, Huifang Yin, Jiangang Hu, Jinfeng Miao, Zhaoguo Chen, Kezong Qi, Zhihao Wang, Jiansen Gong, Vanhnaseng Phouthapane, Wei Jiang, Rongsheng Mi, Yan huang, Chen Wang, Xiangan Han

**Affiliations:** 1 Shanghai Veterinary Research Institute, The Chinese Academy of Agricultural Sciences (CAAS), 518 Ziyue Road, Shanghai, 200241 People’s Republic of China; 2 College of Life Science, Longyan University, Longyan, 364000 People’s Republic of China; 3 College of Animal Science and Technology, Anhui Agricultural University, Hefei, 230036 People’s Republic of China; 4 College of Veterinary Medicine, Nanjing Agricultural University, Nanjing, 210095 People’s Republic of China; 5 Poultry Institute, Chinese Academy of Agricultural Sciences, Yangzhou, 225125 People’s Republic of China; 6 Biotechnology and Ecology Institute, Ministry of Science and Technology (MOST), Vientiane, 22797 Lao PDR; 7 College of Animal Science and Technology, Henan University of Science and Technology, No. 263 Kaiyuan Road, Luoyang, 471023 People’s Republic of China

## Abstract

The function of Autoinducer-2 (AI-2) which acts as the signal molecule of LuxS-mediated quorum sensing, is regulated through the *lsr* operon (which includes eight genes: *lsrK*, *lsrR*, *lsrA*, *lsrC*, *lsrD*, *lsrB*, *lsrF*, and *lsrG*). However, the functions of the *lsr* operon remain unclear in avian pathogenic *Escherichia coli* (APEC), which causes severe respiratory and systemic diseases in poultry. In this study, the presence of the *lsr* operon in 60 APEC clinical strains (serotypes O1, O2, and O78) was investigated and found to be correlated with serotype and has the highest detection rate in O78. The AI-2 binding capacity of recombinant protein LsrB of APEC (APEC-LsrB) was verified and was found to bind to AI-2 in vitro. In addition, the *lsr* operon was mutated in an APEC strain (APEC94Δlsr(Cm)) and the mutant was found to be defective in motility and AI-2 uptake. Furthermore, deletion of the *lsr* operon attenuated the virulence of APEC, with the LD_50_ of APEC94Δlsr(Cm) decreasing 294-fold compared with wild-type strain APEC94. The bacterial load in the blood, liver, spleen, and kidneys of ducks infected with APEC94Δlsr(Cm) decreased significantly (*p* < 0.0001). The results of transcriptional analysis showed that 62 genes were up-regulated and 415 genes were down-regulated in APEC94Δlsr(Cm) compared with the wild-type strain and some of the down-regulated genes were associated with the virulence of APEC. In conclusion, our study suggests that *lsr* operon plays a role in the pathogenesis of APEC.

## Introduction

Avian pathogenic *Escherichia coli* (APEC) infection causes severe respiratory and systemic diseases in poultry, resulting in serious losses to the poultry industry worldwide [1]. Elucidation of the underlying molecular mechanisms of APEC pathogenicity is crucial for controlling avian colibacillosis. The pathogenicity of APEC is determined by many factors, such as virulence factors, secretion systems, and quorum sensing [1–3]. The AI-2 signal molecule of the LuxS/AI-2 quorum sensing system, which is derived from *S*-adenosylmethionine (SAM) by LuxS synthase, is widely distributed among bacteria. Currently, AI-2 is regarded as the most ubiquitous bacterial intercommunication signal and has been reported to play an important role in the regulation of many bacterial behaviors (including survival, biofilm formation, and virulence-related gene expression) [4] via AI-2 receptors.

There are two main types of AI-2 receptors, LuxP and LsrB receptors, which were initially characterized in *Vibrio harveyi* and *Salmonella typhimurium*, respectively. Although both LuxP and LsrB can bind to AI-2 in vitro, these two receptors have different structures [4].

In *S. typhimurium*, *lsrB* gene is located in *lsr* operon, which was reported to function in importing AI-2 into the bacteria [5]. The *lsr* operon consists of eight genes (*lsrKRACDBFG*) that encode an ATP binding cassette (ABC) transporter. LsrK is a kinase that phosphorylates AI-2 and is required for the regulation of AI-2 uptake [6]. LsrR is a repressor of the *lsr* operon and during exponential growth, phosphor-AI-2 could inactivate LsrR and subsequently induce the transport of LsrACDBFG [7]. The *lsrACDB* genes encode the Lsr transport apparatus located in the cell membrane. The *lsrF* gene encodes a coenzyme A-dependent thiolase that catalyzes the final step in the processing of the quorum sensing signal AI-2 [8]. LsrB is one of the substrate binding proteins of the ABC transport system that is responsible for the internalization of extracellular AI-2. Although orthologs of LsrB were found in *S. typhimurium* [9], *E. coli* [10], the plant symbiont *Sinorhizobium meliloti* [11], and the oral pathogen *Aggregatibacter actinomycetemcomitans* [12], in general, orthologs of the Lsr system are not broadly conserved in bacteria. Furthermore, although in *E. coli*, AI-2 is taken up intracellularly by the Lsr system, no typical LsrB-like receptor has been detected in APEC [4], and to date, there has been no report of the presence of a similar Lsr system in APEC strains.

Our previous study found that APEC strain DE17 (serotype O2) possessed the *luxS* gene (but lacked *lsr* operon), and deletion of the *luxS* gene decreased APEC pathogenicity [2]. Interestingly, another APEC strain, APEC94 (serotype O78), was found to possess *lsr*-like operon in this study. These results revealed that *lsr* operon had a different distribution rate in different serotypes APEC strains. Furthermore, *lsr* operon is involved in transportation of AI-2, which may regulate some other physiological functions in bacteria. To date, the role of *lsr* system in APEC strains remains unknown; however, we proposed that the Lsr system may be involved in regulating the virulence of APEC strains. Hence, in this study, we investigated: (1) the *lsr* operon distribution among 60 APEC clinical strains of the three most common serotypes, (2) whether *lsr* operon participates in AI-2 transportation, and (3) whether *lsr* system is involved in regulating the virulence of APEC. Our findings provide a basis for further studies into the pathogenic mechanisms of APEC from a quorum sensing perspective.

## Materials and methods

### Bacterial strains, plasmids, and culture conditions

All of the strains and plasmids used in this study are listed in Table 1. The *E. coli* strains were grown in Lennox broth (LB, Oxoid, UK) or solid medium containing 1.5% agar at 37 °C overnight. *V. harveyi* strains were grown in marine broth (MB, Becton–Dickinson, Sparks, MD, USA) or solid medium containing 1.5% agar at 28 °C. Modified autoinducer bioassay (AB) medium was exclusively used for *V. harveyi* strain BB170 [13] for the detection of bioluminescence. The ingredients of AB were purchased from Amresco, USA. When needed, antibiotics (Sangon Biotech, Shanghai, China) were added to the following final concentrations: 100 μg/mL ampicillin, 30 μg/mL chloramphenicol, and 50 μg/mL kanamycin.

**Table 1:** **Bacterial strains and plasmids used in this study**

Strains or plasmid	Relevant genotype and property	Source or references
*Escherichia coli* strains		
BL21	DE3	Invitrogen
BL21ΔluxS	BL21 DE3*luxS* deletion mutant strain	[23]
MG1655		[16]
DE17	APEC serotype O2	[2]
DE17ΔluxSΔpfs	DE17 *luxS* and *pfs* mutant strain	This study
APEC94	Wide type, isolated from sick chicken (serotype O78)	This study
APEC94Δlsr(Cm)	Lacking of *lsr* operon	This study
*Salmonella* strains		
*Salmonella typhimurium*	LT2	ATCC 14028
*Vibrio harveyi* strains		
BB170	BB120 luxN::Tn5 (sensor 1−, sensor 2 +)	[13]
BB152	BB120 luxM::Tn5 (representing AI-2)	[21]
Plasmids		
pKD46	The vector containing arabinose-inducible phage λ Red recombinase (Amp^R^)	[17]
pACYC177-lsr	The *lsr* operon complement plasmid	This study
pCold TF	Expression vector with Amp^R^	TaKaRa
pKD3	The plasmid contained chloramphenicol resistance (Cm^R^)	[17]
pCold TF-lsrB	Recombinant plasmid contained *lsrB* of APEC	This study

### Ethics statement

According to the guidelines of the Institutional Animal Care and Use Committee (IACUS), one-day-old Cherry Valley ducklings purchased from Shanghai Zhuang Hang Duck Farm were cared for and maintained by the Shanghai Veterinary Research Institute of Chinese Academy of Agricultural Sciences (CAAS). All the animal experiments were approved by the Ethics Committee of CAAS (Permit No: CAAS-SHVRI-Po-2019-36).

### Isolates serotyping and sequencing and alignment of *lsr* operon of APEC94

Sixty clinical isolates of APEC (serotypes O1, O2, O78) were obtained from obviously diseased poultry in the provinces of Shanghai, Anhui, Jiangsu, Fujian, and Henan between 2007 and 2015. The DNA of the clinical isolates was extracted from overnight cultures using a boiling procedure according to a previously reported protocol [14]. The serotypes of APEC strains were determined using a multiplex PCR method as described previously [15]. To investigate the distribution of the *lsr* operon among APEC strains, primers (*lsrK*-F/R, *lsrR*-F/R, *lsrA*-F/R, *lsrC*-F/R, *lsrD*-F/R, *lsrB*-F/R, *lsrF*-F/R, *lsrG*-F/R, Table 2) were designed for the 8 genes from the *lsr* operon according to the corresponding sequences from strain MG1655 [16]. In addition, the PCR products of APEC94 were sequenced (by Invitrogen) and aligned with the corresponding sequences from strain MG1655 and *Salmonella* strain LT2.

**Table 2:** **Primers used in this study**

Primers	Oligonucleotide sequence (5′ to 3′)	Description	Product size (bp)
*lsrK*-F	CTATAACCCAGGCGCTTTCCATA	Partial sequence of *lsrK*	1593
*lsrK*-R	ATGGCTCGACTCTTTACCCTTTC		
*lsrR*-F	TTAACTACGTAAAATCGCCGCTG	Partial sequence of *lsrR*	954
*lsrR*-R	ATGACAATCAACGATTCGGCAAT		
*LsrA*-F	ATGCAAACGAGTGATACCCGCGC	Partial sequence of *lsrA*	1539
*lsrA*-R	ACTTCAGCATGACGCCTCCTGAC		
*lsrC*-F	CTGAAGTTTATTCAGAACAACCGTG	Partial sequence of *lsrC*	826
*lsrC*-R	TATCGATCTGCGTCAGGAACCAT		
*lsrD*-F	GCCGCATAATGCGTATTCGCTAC	Partial sequence of *lsrD*	1003
*lsrD*-R	CTTTATGGCAATGGGTTATTGGC		
*lsrB*-F	ATGACACTTCATCGCTTTAAGA^a^	Partial sequence of *lsrB*	826
*lsrB*-R	CTGAAATTTTGCCTTGCTGAAC^a^		
*lsrF*-F	GTAAAGATTTTCGTACCGATCAAC	Partial sequence of *lsrF*	657
*lsrF*-R	TTTTACCGCCAGCAATAACAATG		
*lsrG*-F	ATGCACGTCACACTGGTTGAAAT	Partial sequence of *lsrG*	291
*lsrG*-R	TCACGGCATCAAACCATTGAACA		
APEC94lsr-F	TCCCTTGAATATCGTACTGG	Using for DNA sequencing of *lsr* operon	
APEC94lsr-R	TGCGCTTCGATGTCTTAC		
pCold-F1	CCCAAGCTTGAAATCGTATTTGCCGAT	Using for identification of *lsrB*-APEC recombinant plasmid	1150
pCold-R	GGCAGGGATCTTAGATTCTG		
*lsr*-UF	TCTAAAAGAAGGGAAATAAG^b^	The upstream sequence of *lsr* operon	763
*lsr*-UR	CCAGCCTACA GCATGGTACAAGCTGAATC		
*lsr*-CF	TGTACCATGCTGTAGGCTGGAGCTGCTT	Chloramphenicol resistance cassette (cat)	1013
*lsr*-CR	CATTGAACAGCATATGAATATCCTCCTTAGTTC		
*lsr*-DF	TATTCATATGCTGTTCAATGGGTTGATGC	The downstream sequence of *lsr* operon	638
*lsr*-DR	TGGTGATAGTAGGTGGTTCG^b^		
PstI-*lsr*-CF	AAAA**CTGCAG**GGTCTGTATTGAGTGTTAGTTGGAGGTGGG^c^	The *lsr* operon complement sequence	9440
PstI-*lsr*-CR	AAAA**CTGCAG**CAGTTGCGGATGTCTGCTT^c^		
*LsrB*-BamHI-F	CGC**GGATCC**ATGACACTTCATCGCTTTA^d^	The ORF sequence of *lsrB*	1023
*LsrB*-HindIII-R	CCA**CTTTCA**ACGAGCTGATG^e^		
*fimH*-RTF	GTGCCAATTCCTCTTACCGTT	Partial DNA sequence of fimbrial protein FimH	165
*fimH*-RTR	TGGAATAATCGTACCGTTGCG		
*fliD*-RTF	TTCAGACGCAGTTGAAATCG	Partial DNA sequence of flagellar filament capping protein FliD	184
*fliD*-RTR	TTACCATCGCCAACAATCAA		
*flhA*-RTF	CGGCATCGTACTCTGGAACT	Partial DNA sequence of flagellar biosynthesis gene *flhA*	172
*flhA*-RTR	CATTACCCATCCGTTCGTTC		
*dosP*-RTF	ATAAACCCACGCCCATATCA	Partial DNA sequence of oxygen-sensing cyclic-di-GMP phosphodiesterase DosP	194
*dosP*-RTR	GGCGTTATCCGTGAACTTGT		
*pgaA*-RTF	GGCAATGGTCTCCTTGTGAT	Partial DNA sequence of poly-beta-1,6 *N*-acetyl-d-glucosamine export porin PgaA	195
*pgaA*-RTR	GATCATCTTGGCGCGTTATT		
*fhuD*-RTF	AACTATCGCCTGTGGGTCAG	Partial DNA sequence of iron-hydroxamate transporter substrate-binding subunit fhuD	153
*fhuD*-RTR	TGCCAGCATTTCTGATGAAG		
*dnaE*-RTF	GATTGAGCGTTATGTCGGAGGC	Partial DNA sequence of the internal control *dnaE*	80
*dnaE*-RTR	GCCCCGCAGCCGTGAT		

^a^A 826-bp PCR product was amplified from wild-type strain APEC94 but no PCR product was obtained from mutant strain APEC94Δlsr(Cm) using primers *lsrB*-F/*lsrB*-R.

^b^A 2414-bp PCR product was amplified from mutant strain APEC94Δlsr(Cm) but no product was amplified from wild-type strain APEC94 in a limited amplification time using primers *lsr*-UF/*lsr*-DR.

^c^PstI restriction sites are underlined.

^d^BamHI restriction sites are underlined.

^e^HindIII restriction sites are underlined.

### Construction and identification of lsr mutant strain and its complementation of APEC94

The mutant strain APEC94Δlsr(Cm) was generated using the lambda Red recombinase method [17]. Briefly, the upstream and downstream fragments of the *lsr* operon were amplified from the APEC94 genome by PCR using the primers *lsr*-*UF*/*lsr*-*UR* and *lsr*-*DF*/*lsr*-*DR*. The chloramphenicol resistance cassette (Cm) was amplified from plasmid pKD3 with primers *lsr*-*CF*/*lsr*-*CR*. All of these PCR products (including the upstream and downstream fragments and the Cm cassette) were then used as templates for amplifying the recombinant fragment with primers *lsr*-*UF*/*lsr*-*DR* by overlap PCR. One microgram of the recombinant PCR product was then added to 100 μL of APEC94 competent cells containing the lambda Red recombinase expression plasmid pKD46. Electroporation was performed as described previously. Positive clones were selected on LB plates containing 10 μg/mL chloramphenicol and were verified by PCR using primer pairs *lsr*-*UF*/*lsr*-*DR* and *lsrB*-*F*/*lsrB*-*R*.

For functional complementation, *lsr* operon sequence (containing 9440 bp) was amplified from the chromosomal DNA of APEC94 with the primers PstI-*lsr*-CF/PstI-*lsr*-CR and cloned into pACYC177 to yield the plasmid pACYC177-lsr.

The mutant strain APEC94Δlsr(Cm) was transformed with pACYC177-lsr. The complementation strain was named *lsr*-APEC94Δlsr. The primer sequences used are listed in Table 2.

### The effect of lsr operon on growth, biofilm formation and motility of APEC94

Growth curves were determined as described previously [18]. Briefly, equal volume (1 mL) of APEC94, APEC94Δlsr(Cm) and *lsr*-APEC94Δlsr culture (OD_600_ = 1.0) were inoculated into 100 mL of LB culture media and incubated at 37 °C with shaking, respectively. The OD_600_ value of each sample was then examined at 1 h intervals. The ability of biofilm formation was assessed by crystal violet staining method. The bacteria were cultured to a logarithmic phase and centrifuged at 4 °C, 6000 rpm. Then the cells were resuspended with the M9 medium (containing 0.2% fructose) after washing twice with PBS and adjusted to OD_600_ = 0.1. The bacteria were at incubated at 25 °C for 18 h in a sterile 96-well plate (200 μL per well and each group for 5 repeats), then washed with PBS (200 μL per well), and stained with 0.1% crystal violet at 37 °C for 15 min. The plates were washed with PBS for three times and dried. Finally, 95% ethanol was added into the wells and the OD_595_ was measured using microplate reader to analyze the biofilm formation of APEC94, APEC94Δlsr(Cm) and lsr-APEC94Δlsr, respectively.

The swimming motility of APEC94, APEC94Δlsr(Cm) and *lsr*-APEC94Δlsr bacterial cells was assessed on soft agar plates (1% tryptone, 0.8% glucose, 0.5% NaCl, 0.3% agar) as described previously with some modifications [19]. Briefly, the bacteria were cultured to logarithmic phase and pelleted by centrifugation. The resulting pellet was washed and suspended in phosphate-buffered saline (PBS) and the final OD_600_ was adjusted to 1.0. A total of 6 μL of bacterial suspension was loaded onto the soft agar plate and incubated for 24 h. The swimming motility was assessed by measuring the migration diameter of bacteria from the center toward the periphery on the agar plate.

### AI-2 bioassay

The detection of AI-2 activity was performed according to a previously described method [3, 20]. Briefly, cell-free culture fluid (CF) of APEC strains and mutants was prepared as follows. Bacteria were cultured in LB at 37 °C and pelleted by centrifugation at 12 000 *g* at 4 °C for 10 min. Then, CF samples were obtained by filtering the resulting supernatants via a 0.22-μm filter (EMD Millipore, Bedford, MA, USA). The reporter strain *V. harveyi* BB170 was diluted to 1:5000 in fresh AB medium. Then, 20 μL of the bacterial CF sample to be tested was added to 180 μL of bacterial culture and incubated at 30 °C for 4 h. After incubation, 200-μL aliquots were added to white, flat-bottomed, 96-well plates (Thermo Labsystems, Franklin, MA, USA) for the detection of AI-2 activity. A positive control was obtained from overnight cultures of BB152 [21], while the CF from DE17ΔluxSΔpfs strain (constructed in our laboratory) was used as a negative control. Luminescence was measured with a microplate reader in luminescence mode (Synergy2, BioTek, USA). The AI-2 activity of *V. harveyi* BB170 is expressed in relative light units (RLU). All samples were assayed in triplicate.

### Determination of the AI-2 internalization curve

AI-2 internalization curves of APEC94 and APEC94Δlsr(Cm) were determined as described previously [22]. Briefly, APEC94 and APEC94Δlsr(Cm) were cultured to logarithmic phase and the bacteria were harvested via centrifugation. Bacteria were resuspended in PBS, washed twice, and then the OD_600_ was adjusted to 1.0. The CF samples were obtained by filtration of the resulting supernatant through a 0.22-μm filter to assess the endogenous AI-2 levels of APEC94 and the mutant strain.

To generate AI-2 internalization curves for strains APEC94 and APEC94Δlsr(Cm), 10 mL of bacterial culture with a final concentration of 36 μM commercial AI-2 was incubated at 37 °C with shaking. The CF samples of each strain were obtained as described previously every 2 h. The CF from BB152 was used as the positive control and the CF from the DE17 *luxS* and *pfs* double mutant strain was used as the negative control (Table 1).

The detection of AI-2 activity was performed as described previously. The AI-2 activity of *V. harveyi* BB170 is expressed in RLU. All samples were assayed in triplicate.

### Expression of LsrB and AI-2 binding assays

To determine whether the LsrB of APEC94 can bind to AI-2, the *lsrB* gene was expressed in *E. coli* strains BL21 and BL21ΔluxS [23]. Recombinant LsrB fusion proteins were purified by Ni-chelating affinity chromatography after ultrasonic decomposition and eluted using a solution containing 50 mM imidazole. The LsrB fusion proteins were investigated by SDS-PAGE gel scan analysis.

An AI-2 release assay was performed according to a previously described method with some modifications [23]. The purified LsrB fusions (3 mg/mL) isolated from BL21 or BL21ΔluxS strains were incubated for 10 min at 55 °C to release endogenous AI-2. After incubation, the LsrB proteins were removed by ultrafiltration (10 000-Da cut-off; EMD Millipore), and the filtered reaction products were tested for AI-2 activity using a *V. harveyi* BB170 bioassay, performed as described above. Sterile PBS with commercial AI-2 was used as a positive control.

An assay of the binding of recombinant LsrB to exogenous AI-2 was performed according to a previously described method with some modifications [23]. Purified LsrB fusions (3 mg/mL) isolated from strains BL21 or BL21ΔluxS were incubated with exogenous AI-2 (40 μM) for 1 h at 37 °C. After incubation, the LsrB fusion proteins were removed by ultrafiltration (10 000-Da cut-off; EMD Millipore), and the filtered reaction products were tested for AI-2 activity using a *V. harveyi* BB170 bioassay as detailed above. Sterile PBS with commercial AI-2 was used as a positive control.

### Determination of the lethal dose of APEC94 and APEC94Δlsr(Cm)

To verify whether deletion of the *lsr* operon would influence the virulence of APEC strains, 9-day-old Cherry Valley ducklings were used to determine the LD_50_ of the bacteria as described previously [24]. Briefly, for APEC94 and APEC94Δlsr(Cm), 40 ducklings of each group were divided evenly into five groups and injected intramuscularly into legs with 10^9^, 10^8^, 10^7^, 10^6^, or 10^5^ CFU of bacteria, respectively. Mortality was monitored until 7 days post-infection and the LD_50_ was calculated according to the Reed–Muench formula [25].

### The bacterial loads of APEC94 and APEC94Δlsr(Cm) in vivo

The bacteria strains were grown to an OD_600_ value of 1.0, washed three times using sterile PBS, and subsequently suspended in PBS. For each group, eight 9-day-old Cherry Valley ducklings were injected with 10^9^ CFU of bacteria. Ducklings were euthanized and dissected 24 h post-infection. The bacterial loads of each sample in the blood, liver, spleen, and kidney of the infected ducklings were counted by the plate dilution method as described previously [1].

### Collection of bacterial samples for transcriptional analysis

The transcriptional levels of APEC, APEC94 and APEC94Δlsr(Cm) cells were determined as described with some modification [26]. To evaluate the effects of the *lsr* operon on the transcriptional levels of APEC, APEC94 and APEC94Δlsr(Cm) cells were collected from culture in LB medium when the OD_600_ was 1.0 by centrifugation at 12 000 rpm for 5 min. The collected cells were washed with PBS (pH = 7.4) and centrifuged. Total RNA was extracted from the bacterial cell pellet using Trizol RNA Isolation Reagent (Invitrogen, Carlsbad, CA, USA) according to the manufacturer’s protocol. RNA quantity and quality were evaluated by the OD_260_/OD_280_ ratio using a Nanodrop spectrophotometer. RNA integrity was assessed by standard denaturing agarose gel electrophoresis [26]. mRNA was enriched by removing rRNA from the total RNA samples using the Ribo-zero kit. Then, the transcriptome libraries of APEC94 and APEC94Δlsr(Cm) were constructed and sequenced using the Illumina HiSeqTM2500/MiseqTM sequencing technique. The main differentially expressed genes were analyzed by gene ontology (GO). Pathway analysis for differentially expressed genes was performed according to the latest Kyoto Encyclopedia of Genes and Genomes (KEGG) database. The readcount obtained was firstly normalized according to the method of TMM (Trimmed mean of M-values) and the *p* value was calculated abided by the Poisson distribution. Differentially expressed genes were defined by a fold change in expression of > 2 and q value < 0.005. Volcano Plot filtering was performed to identify the statistically significant differentially expressed genes between APEC94 and APEC94Δlsr(Cm).

### The effect of *lsr* operon on transcriptional levels of virulence-related genes

To analyze the effect of *lsr* operon on the expression of the genes involved in virulence of APEC *fimH*, *fliD*, *flhA*, *dosP*, *pgaA* and *fhuD* [27–32], their expression was evaluated by quantitative real time-PCR (Table 2).

Briefly, bacteria were grown to the logarithmic phase (OD_600_ = 1.0) in LB broth and total RNA was extracted from these bacteria using TRIzol reagent (Invitrogen Corporation, USA) according to the manufacturer’s protocol. The RNA was treated with PrimeScript™ RT reagent Kit with gDNA Eraser (TAKARA Corporation, Japan) in order to remove genomic DNA, and then reverse transcribed into cDNA (complementary DNA). The SYBR Green based two-step qRT-PCR (TB Green^®^ Premix Ex Taq™ II, TAKARA Corporation, Japan) amplification was performed as described previously with some modification. Relative changes in gene expression level were assessed using the 2^−∆∆Ct^ method [2]. The *dnaE* gene was used as an internal control. The final qRT-PCR data were presented as the means of three separate experiments.

## Results

### Distribution of the *lsr* operon between different serotype strains

The PCR assay results confirmed that 41 of the 60 APEC isolates (68.3%) possessed *lsr* operon. Interestingly, among these 41 isolates, five possessed an incomplete *lsr* operon.

The distribution of *lsr* operon was found to be different for different serotypes with positive detection rates in serotypes O1, O2, and O78 of 68.8% (11/16), 40.9% (9/22), and 95.5% (21/22), respectively. There was one isolate with an incomplete *lsr* operon in serotype O1, two isolates in serotype O2, and two isolates in serotype O78, respectively (Additional file 1).

### Analysis of the *lsr* operon in APEC94

According to nucleotide sequence alignments, the eight genes in the *lsr* operon of APEC94 shared high homology to MG1655 (> 98%), and 66–79% homology to *Salmonella* strain LT2 (Additional file 2).

According to amino acid sequence alignments, the proteins encoded by the eight genes in the *lsr* operon of APEC94 shared high homology to MG1655 (> 99%), and 71–89% homology to *Salmonella* strain LT2 (Additional file 3).

### Identification of *lsr* operon mutant and complementary strains of APEC94, analysis of the effect of *lsr* operon on the characteristics of APEC94

**Figure 1:**
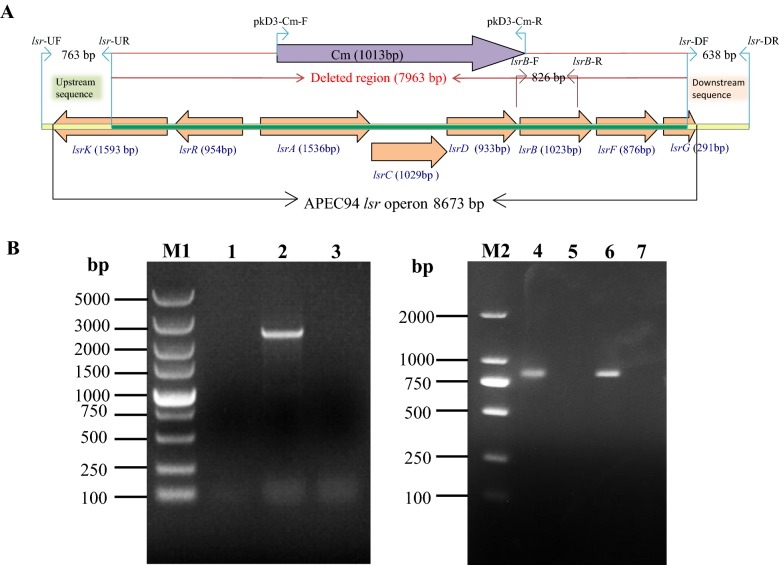
**PCR analysis of the** ***lsr*** **mutant strain APEC94Δlsr(Cm) and** ***lsr*** **complementary strain** ***lsr*-APEC94Δlsr. A** Schematic chart of the strategy for producing the *lsr* operon deletion mutant. The *lsr* operon was deleted by replacing the partial gene sequence with a chloramphenicol resistance cassette. The primers used for confirmation of the *lsr* deletion are also indicated. **B** Identification of the *lsr* mutant strain APEC94Δlsr(Cm) and *lsr* complementary strain *lsr*-APEC94Δlsr. M1: 5000 DNA marker; M2: 2000 DNA marker; Lane 1: The wild-type strain APEC94 showed no PCR product using primers *lsr*-UF/*lsr*-DR because the amplification time was limited beyond the amplification efficiency of DNA polymerase; Lane 2: The mutant strain APEC94Δlsr(Cm) showed a 2414-bp PCR product using primers *lsr*-UF/*lsr*-DR, which amplified upstream and downstream sequences and the chloramphenicol resistance cassette; Lane 3: Negative control; Lane 4: The wild-type strain APEC94 showed a 826-bp PCR product using primers lsrB-F/lsrB-R; Lane 5: The mutant strain APEC94Δlsr(Cm) showed no PCR product using primers *lsrB*-F/*lsrB*-R; Lane 6: The *lsr* complementary strain *lsr*-APEC94Δlsr showed a 826-bp PCR product using primers *lsrB*-F/*lsrB*-R; Lane 7: Negative control.

**Figure 2:**
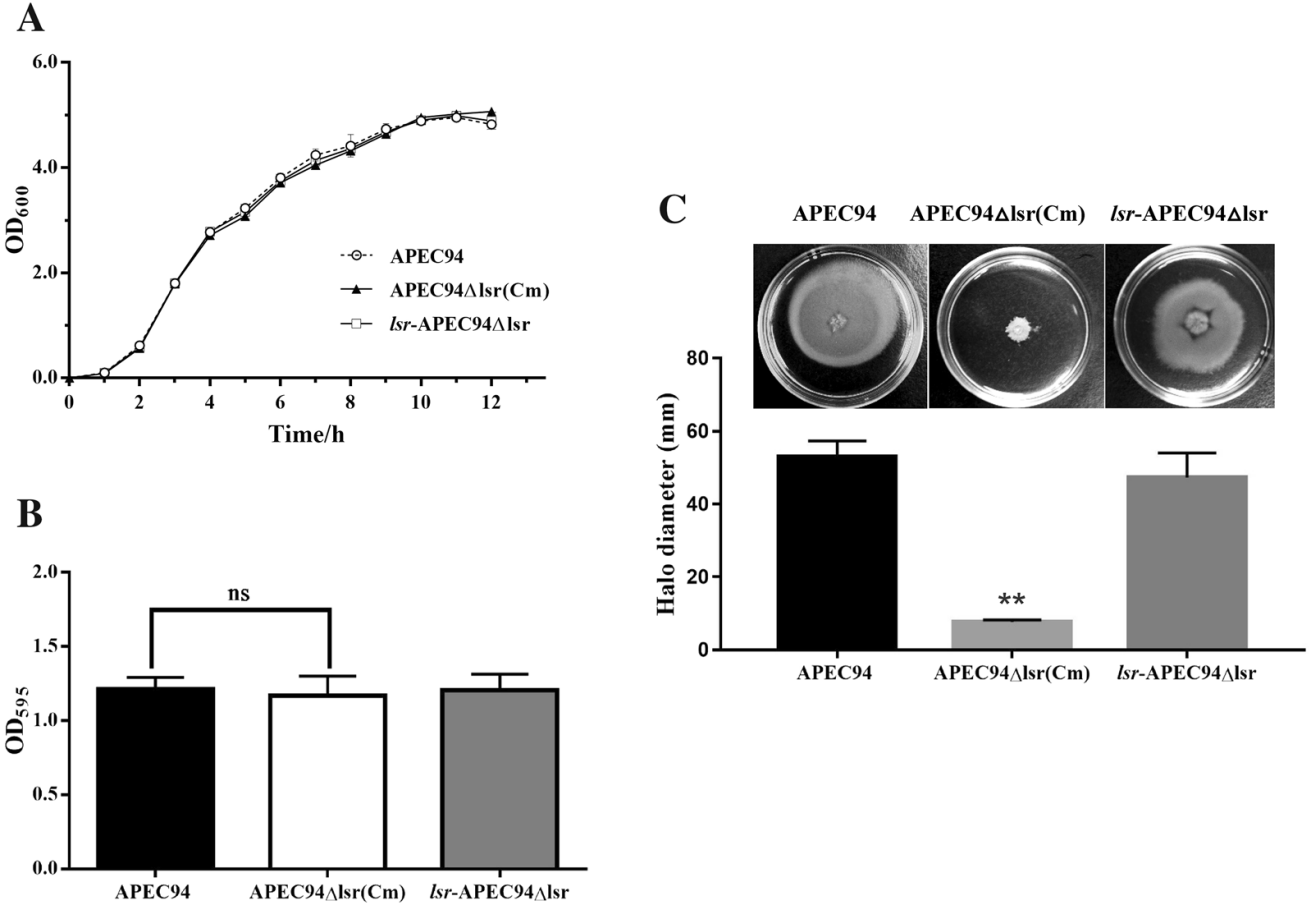
**Characteristics of strains APEC94, APEC94Δlsr(Cm) and lsr-APEC94Δlsr. A** Growth curves of APEC94, APEC94Δlsr(Cm) and *lsr*-APEC94Δlsr in LB. The growth rates of APEC94, APEC94Δlsr(Cm) and *lsr*-APEC94Δlsr were monitored by measuring their OD_600_ values in LB medium at 1-h intervals for 12 h, respectively. After 12 h of culturing, there was no significant difference in the growth rate of APEC94, APEC94Δlsr(Cm) and lsr-APEC94Δlsr grown in LB broth (*p* > 0.05). Each value represents the average of three independent experiments. Significant differences were detected using the Student’s *t* test with SPSS v19.0 software. **B** Biofilm formation. There was no significant difference in biofilm formation among APEC94, APEC94Δlsr(Cm) or *lsr*-APEC94Δlsr strains (*p* > 0.05). All assays were performed in triplicate and repeated three times. *p*-values of less than 0.05 were considered significant. **C** Bacterial motility. The swimming motility of strain APEC94Δlsr(Cm) was significantly lower than that of the wild-type APEC94 strain (*p* < 0.01) while the swimming motility of complementary strain *lsr*-APEC94Δlsr was restored. The experiment was repeated three times and all samples were measured in triplicate. *p* values less than 0.05 were considered significant.

The deletion of the *lsr* operon in mutant strain APEC94Δlsr(Cm) was confirmed by PCR (Figure 1A). A 2414-bp PCR product was amplified from the mutant strain APEC94Δlsr(Cm) with primers *lsr*-UF/*lsr*-DR (Figure 1B, lane 2), whereas no product was obtained from the wild-type strain APEC94 within a limited amplification time (Figure 1B, lane 1). When primers *lsrB*-F/*lsrB*-R were used, an 826-bp PCR product was amplified from the wild-type strain APEC94 (Figure 1B, lane 4) and *lsr* complementary strain *lsr*-APEC94Δlsr (Figure 1B, lane 6), whereas no PCR product was amplified from APEC94Δlsr(Cm) (Figure 1B, lane 5). Growth curves of APEC94, *lsr* mutant strain APEC94Δlsr(Cm) and *lsr* complementary strain *lsr*-APEC94Δlsr (Figure 2A) showed that there was no significant difference in growth during culture in LB (*p* > 0.05). The crystal violet staining results (Figure 2B) showed that *lsr* operon did not affect biofilm formation of APEC94, APEC94Δlsr(Cm) and *lsr*-APEC94Δlsr (*p* > 0.05). As shown in Figure 2C, compared with the wild-type strain, the motility of APEC94Δlsr(Cm) was significantly decreased (*p* < 0.01) while the motility of complementary strain *lsr*-APEC94Δlsr was restored (*p* > 0.05).

### The *lsr* operon influenced the uptake of AI-2 in APEC94

**Figure 3:**
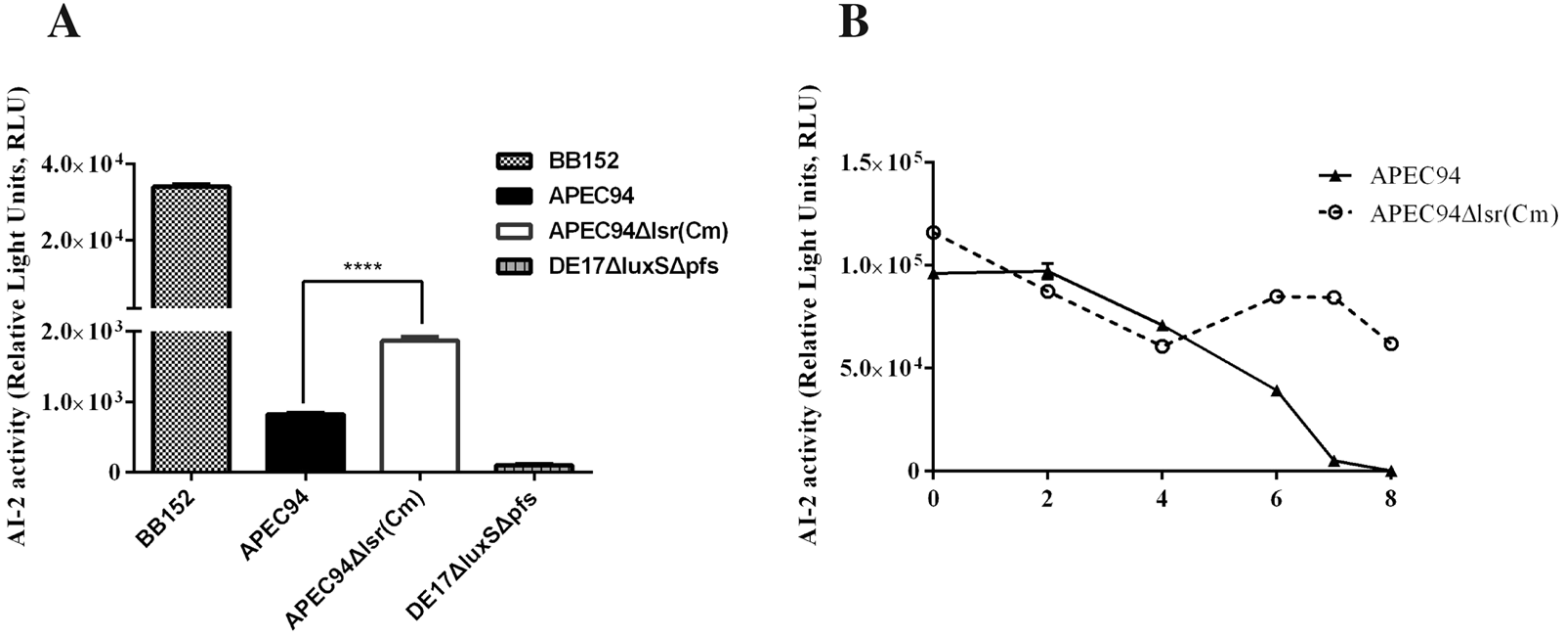
**Influence of the** ***lsr*** **operon on the bacterial AI-2 internalization curve. A** The AI-2 activity of bacterial cell-free culture fluids was monitored by an AI-2 bioassay. APEC94 and APEC94Δlsr(Cm) produced AI-2 at the logarithmic phase, but the AI-2 activity of APEC94Δlsr(Cm) (1864 RLUs) was 2.26-fold higher than the value of APEC94 (823 RLUs) (*p* < 0.05). **B** In the AI-2 internalization curve of APEC94, the activity of AI-2 began to decrease rapidly at 4 h and at 8 h the AI-2 activity was nearly negative, while the activity of AI-2 in APEC94Δlsr(Cm) cell suspensions remained high during the 8 h (*p* < 0.001). All samples were measured in triplicate. *p* values less than 0.05 were considered significant.

The CF from APEC94 and APEC94Δlsr(Cm) at logarithmic phase tested positive in a bioluminescence detection assay. However, the RLU value of APEC94Δlsr(Cm) was 2.26-fold higher than that of the wild-type strain (Figure 3A).

When 37.0 μM AI-2 was added to the APEC94 cell suspensions, AI-2 activity was rapidly reduced after 4 h, reaching almost negative control levels by 8 h. By contrast, when 37.0 μM AI-2 was added to the APEC94Δlsr(Cm) cell suspensions, AI-2 activity was maintained at a high level throughout the 8 h (Figure 3B). The results indicated that APEC94 could generate an AI-2-like molecule, the *lsr* operon could influence the uptake of AI-2 in APEC94, and deletion of the *lsr* operon could impede AI-2 internalization.

### LsrB could bind to both endogenous and exogenous AI-2

The recombinant fusion protein APEC-LsrB was successfully expressed in soluble form in both *E. coli* BL21 and BL21∆luxS strains. The fusion proteins were purified and the concentrations in strain BL21(DE3) and BL21∆luxS (DE3) were 0.52 mg/mL and 1.18 mg/mL, respectively.

**Figure 4:**
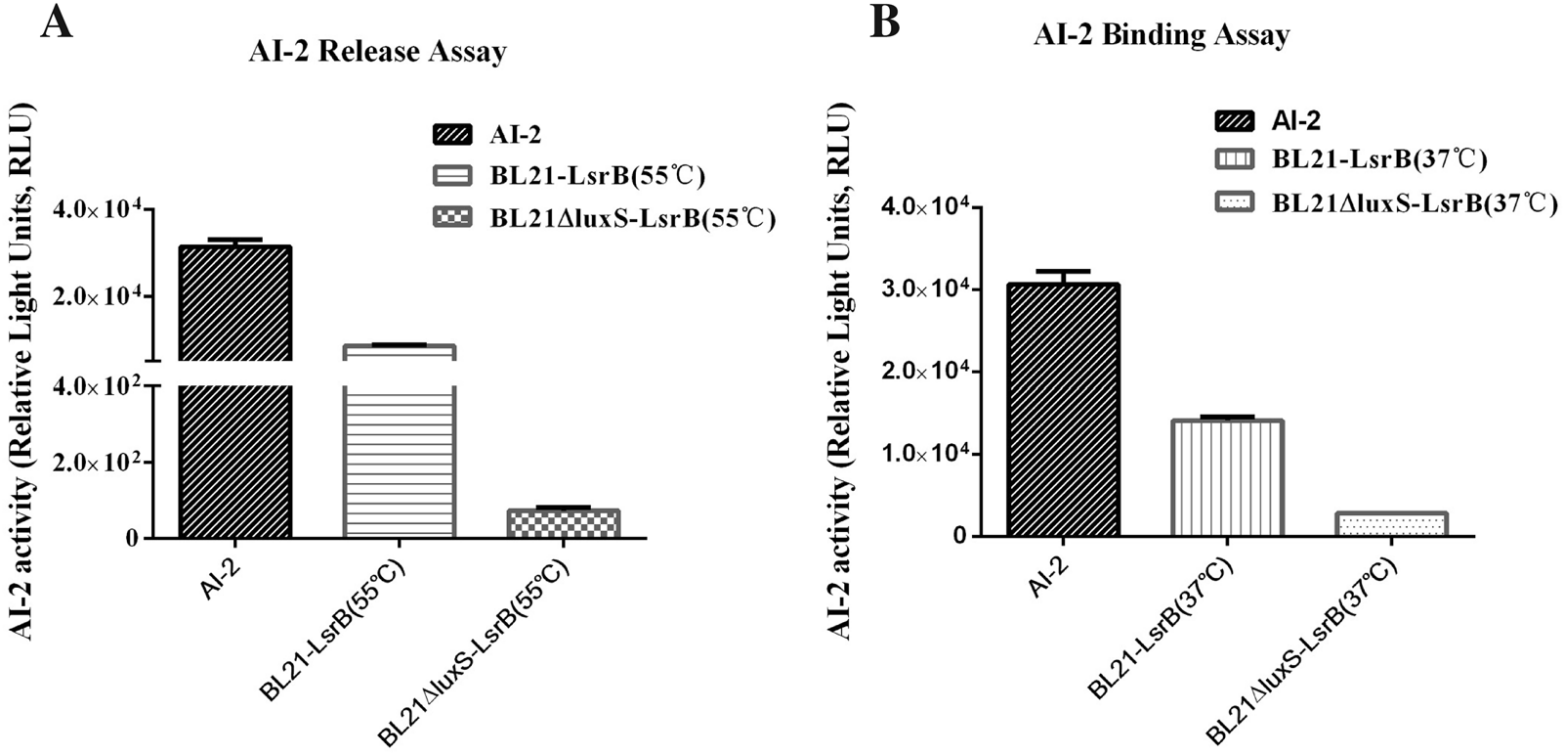
**Binding of APEC-LsrB to AI-2 using an AI-2 assay. A** The activity of APEC-LsrB binding to endogenous AI-2 was evaluated by an AI-2 assay, which showed that the recombinant APEC-LsrB (BL21) bound to endogenous AI-2 produced by wild-type BL21 and released AI-2 at 55 °C. However, the *luxS* mutant BL21∆luxS did not produce AI-2 so that no AI-2 could be released from APEC-LsrB (BL21∆luxS). **B** The activity of APEC-LsrB binding to exogenous AI-2 was evaluated by an AI-2 assay, which showed that APEC-LsrB (BL21∆luxS) could bind to exogenous AI-2, resulting in low AI-2 activity in cell-free culture fluid (CF). However, APEC-LsrB (BL21) could not bind to exogenous AI-2 as it was hindered by endogenous AI-2 (produced by wild-type BL21), which resulted in high AI-2 activity in CF.

The results of the AI-2 release assay showed that recombinant APEC-LsrB (BL21) bound to endogenous AI-2 (produced by the wild-type *E. coli* strain BL21) and released AI-2 after protein denaturation at 55 °C (Figure 4A). However, recombinant APEC-LsrB (BL21∆luxS) could not release endogenous AI-2 affecting the AI-2 synthesis pathway.

An AI-2 binding assay revealed that 3 mg/mL of recombinant APEC-LsrB (BL21∆luxS) could bind to exogenous AI-2 (40 μM), whereas recombinant APEC-LsrB (BL21) could not bind to exogenous AI-2 (40 μM) since it had already bound to endogenous AI-2 (Figure 4B).

### Deletion of the *lsr* operon reduced bacterial virulence

As shown in Table 3, the LD_50_ values of the wild-type and mutant strains were determined to be 8.66 × 10^5^ CFU and 2.55 × 10^8^ CFU, respectively, representing a 294-fold decrease in virulence for the mutant strain APEC94Δlsr(Cm) compared with the wild-type strain.

**Table 3:** **The LD**_**50**_ **of strains APEC94 and APEC94Δlsr(Cm)**

APEC strains	Challenge dosage	No. of ducks	Mortality	Living No.	Mortality rate (%)	LD_50_ (CFU)
APEC94	10^9^	8	8	0	100	8.66 × 10^5^
	10^8^	8	7	1	87.5	
	10^7^	8	7	1	87.5	
	10^6^	8	4	4	50	
	10^5^	8	2	6	25	
APEC94Δlsr(Cm)	10^9^	8	7	1	87.5	2.55 × 10^8^
	10^8^	8	2	6	25	
	10^7^	8	1	7	12.5	
	10^6^	8	0	8	0	
	10^5^	8	0	8	0	

**Figure 5:**
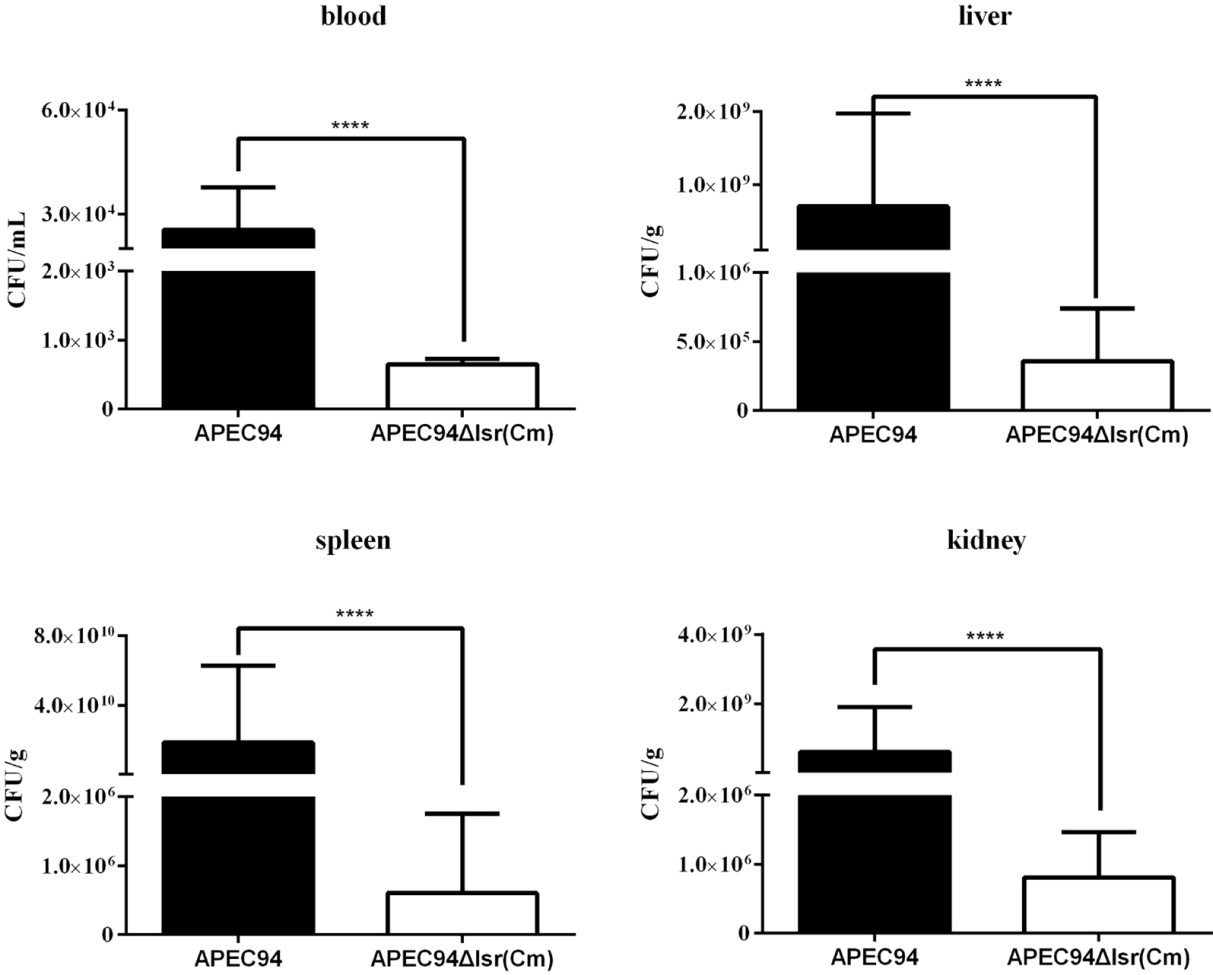
**Bacterial survival in vivo.** The bacterial loads in the blood, liver, spleen, and kidneys were counted. The data are reported per milliliter of blood, and per gram of liver, spleen, and kidney from the infected ducklings. The results showed that the bacterial loads of strain APEC94Δlsr(Cm) in all of the samples were significantly lower than those infected with wild-type strain APEC94, being decreased by 38.5-, 1639-, 316 667-, and 753-fold, respectively (*p* < 0.0001).

The bacterial loads of APEC94Δlsr(Cm) in infected blood, liver, spleen, and kidney were significantly lower than those infected with the wild-type strain APEC94, being decreased by 38.5-, 1639-, 316 667-, and 753-fold, respectively (Figure 5 and Table 4). Statistical analysis of the results using the SPSS analysis software showed that the bacterial loads of APEC94Δlsr(Cm) in all samples were significantly decreased as a result of deletion of the *lsr* operon compared with the wild-type strain APEC94 (*p* < 0.0001).

**Table 4:** **Bacterial loads in ducks**

Tissue and organ	APEC strains		Fold change
	APEC94	APEC94Δlsr(Cm)	
Bacteria in blood (CFU/mL)	2.5 × 10^4^	6.5 × 10^2^	38.5↓
Bacteria in liver (CFU/g)	5.9 × 10^8^	3.6 × 10^5^	1639↓
Bacteria in spleen (CFU/g)	1.9 × 10^10^	6.0 × 10^5^	316 667↓
Bacteria in kidney (CFU/g)	6.1 × 10^8^	8.1 × 10^5^	735↓

“↓” means the fold change of bacterial loads of APEC94Δlsr(Cm) in infected animals decreased compared with APEC94.

### Determination of differentially expressed genes and pathway analysis

**Figure 6:**
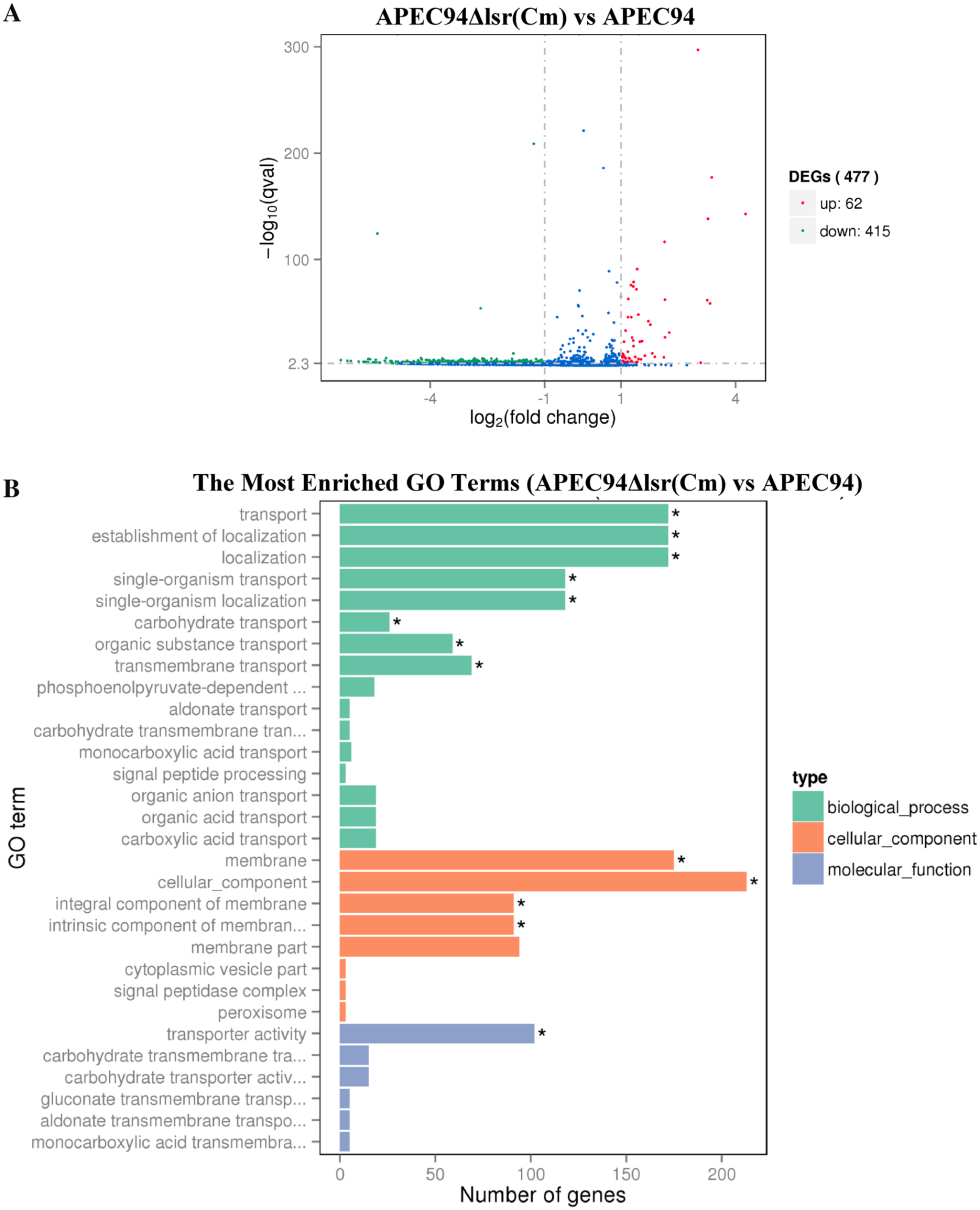
**Differential gene expression. A** Volcano plots were used to visualize differential expression between APEC94 and APEC94Δlsr(Cm). Genes with significantly up-regulated expression are indicated with red dots, down-regulated genes are indicated with green dots, and blue dots represent genes with no significant differential expression. The results showed that 62 genes were up-regulated and 415 genes were down-regulated in APECΔlsr(Cm), compared with wild-type strain APEC94 (differentially expressed genes were selected when the fold change was > 2 and the q value < 0.005). **B** The differentially expressed genes (including up-regulated and down-regulated genes) directly reflected the number and distribution of differentially expressed genes by GO term enriched from three categories: biological progress, cellular component and molecular function. *Represents significantly enriched (*p* < 0.05).

The results of transcriptional analysis showed that 62 genes were up-regulated and 415 genes were down-regulated in APECΔlsr(Cm) compared with wild-type strain APEC94 (Figure 6A). The up- and down-regulated genes were analyzed from three categories: biological process, cellular component, and molecular function (Figure 6B). The most enriched GO terms were associated with the localization and transport of different substances. Some terms could influence the components of the cellular membrane. Moreover, the number of down-regulated genes was significantly higher than that of up-regulated genes.

### The effect of *lsr* operon on transcriptional profiles of virulence-related genes

**Figure 7:**
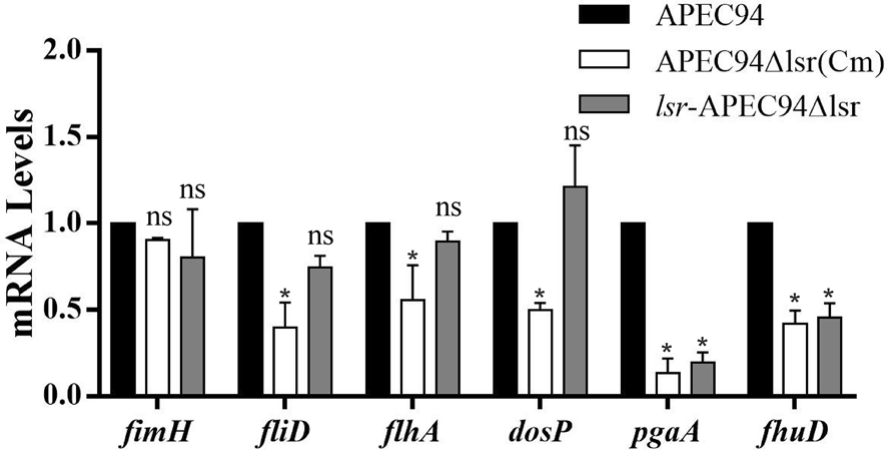
**The effect of** ***lsr*** **operon on transcriptional profiles of virulence-related genes.** The qPCR results showed that there was no significant difference of *fimH* mRNA level between *lsr* mutant strains and APEC94 or *lsr*-APEC94Δlsr (*p* > 0.05). However, the mRNA levels of five genes (*fliD*, *flhA*, *dosP*, *pgaA* and *fhuD*) were significantly decreased in *lsr* mutant strain, as compared to APEC94 (*p* < 0.05).

The qPCR results showed that the mRNA levels of five genes (i.e., *fliD*, *flhA*, *dosP*, *pgaA* and *fhuD*) were significantly decreased in *lsr* mutant strain, as compared to APEC94 (*p* < 0.05). However, *pgaA* and *fhuD* mRNA levels did not restore in *lsr* complementation strain (*p* < 0.05). There was no significant difference of *fimH* mRNA level between *lsr* mutant strains and APEC94 (*p* > 0.05) (Figure 7).

## Discussion

There is increasing awareness of the importance of quorum sensing in coordinating the cooperative behavior of bacteria in response to cell density. AI-2 is regarded as an inter-species communication molecule and its synthesis enzyme, LuxS, is widely distributed among bacteria.

Despite the large number of bacteria in which AI-2 has been detected, only two types of regulation mechanism for AI-2 internalization have been reported in *Vibrio* spp., *Salmonella*, and *E. coli*. In *Vibrio* spp., the *lux* operon is involved in the response to signal transduction of AI-2 and LuxPQ-type of AI-2 receptor has only been found in *Vibrio* spp. [33]. The class of AI-2 receptors are periplasmic two-component sensor kinases and it is only the signal not the AI-2 molecule itself that is transduced intracellularly [33]. In *Salmonella*, the Lsr system is responsible for transferring AI-2 intracellularly and the LsrB-like AI-2 receptor is exposed on the cell surface [10]. This class of AI-2 receptors mainly found in the Enterobacteriaceae, Pasteurellaceae, Rhizobiaceae, and Bacillaceae, but are not detected in APEC [4].

The Lsr operon has been shown to be the transport system for AI-2 in *E. coli*. LuxS was also detected in APEC in our previous studies. However, Pereira et al. reported that no similar Lsr transport system was able to be detected in APEC [4]. This was in agreement with our previous study in one serotype O2 APEC strain DE17, which could internalize AI-2. Furthermore, in the present study, the *lsr* operon genes were not detected in some APEC strains. However, interestingly, the *lsr* operon was identified in APEC94. To analyze the connection between the *lsr* operon distribution and O-antigen serotypes, we detected the presence of the *lsr* operon in the main serotypes (O1, O2, and O78) of APEC. Our findings supported our hypothesis that the *lsr* operon is only present in some APEC strains, although it was present in all three serotypes (O1, O2 and O78). The highest prevalence (95.5%) of the *lsr* operon was detected among serotype O78 strains, followed by serotype O1 strains (68.8%), and serotype O2 strains (40.9%). This may show the divergence between serotypes that has occurred during the evolution of APEC. Five strains were detected with incomplete operons, potentially caused by mutagenesis during the process of evolution. However, the evolutionary basis for these genetic differences requires further study.

In the current study, the *lsr* operon was unable to be amplified from 19 APEC strains, which represented a comparatively high frequency (31.7%) of the strains tested. As APEC was able to remove AI-2 from the culture medium in previous studies, it was proposed that another transport system may be functioning in AI-2 internalization [2]. It was also proposed that some low-affinity transport system may also be operating to internalize AI-2 in *E. coli*, as *E. coli lsrCDB* mutants are still capable of slowly removing AI-2 from culture fluids [10]. Recently, our studies showed that the *ptsI* gene encodes enzyme I, which participates in the phosphotransferase system (PTS) that regulates virulence and AI-2 internalization in APEC [22]. The existence of a new type of AI-2 transport system in APEC and other species is still under investigation and its discovery could potentially offer a therapeutic alternative to antibiotics.

LsrB, encoded by one of the *lsr* operon genes, was first shown to be the receptor for AI-2 in *S. typhimurium*. Orthologs of LsrB were then successively characterized in other bacteria and shown to have AI-2 binding activity [4]. In this study, to assess whether LsrB of APEC has AI-2 binding activity, the *lsrB* gene was expressed in both BL21 and BL21ΔluxS strains. Our previous studies showed that strain BL21(DE3) could generate endogenous AI-2, which interfered with LsrB binding or the release of AI-2. However, the *luxS* mutant of strain BL21 could not produce AI-2 and no sign of interference with LsrB binding and the release of AI-2 was observed. The present study also showed that LsrB of APEC had AI-2 binding activity.

The Lsr system is involved in mediating bacterial uptake of AI-2 from the extracellular environment in many bacteria including *Salmonella typhi* and *E. coli* [4, 6]. In the *lsr* operon, phosphorylated AI-2 is produced by the LsrK protein, along with the consumption of ATP, and a *lsrK* mutant would be defective in the importation of AI-2 from culture fluids. The regulation of the *lsr* operon by transport, phosphorylation and processing of phosphor-AI-2 proceeds through the inactivation of the LsrR repressor [4]. In both *S. typhimurium* and *E. coli*, LsrB was shown to be the receptor for AI-2. To further determine the roles of the *lsr* operon in the transport of AI-2 in APEC, the *lsr* operon was deleted to generate mutant strain APEC94Δlsr(Cm), which was defective in the AI-2 internalization pathway. Thus, in exponential phase, defects in transport meant that AI-2 levels were greater with mutant strain APEC94Δlsr(Cm) than for the wild-type strain. The AI-2 internalization curves of APEC94 and APEC94Δlsr(Cm) further confirmed the role of the *lsr* operon in the uptake of AI-2, and the deletion of *lsr* inhibited AI-2 internalization.

The LuxS/AI-2 quorum sensing system has been shown to regulate bacterial behavior including biofilm formation and virulence. Bacterial motility plays an important role in many aspects of bacterial pathogenicity [34] and has been found to involve several genes, such as the flagellar biosynthesis genes in *Helicobacter pylori*, the biofilm formation genes in MG1655, and luxS in APEC [1, 35, 36]. In this study, mutation of the *lsr* operon significantly decreased the motility of APEC94 on soft agar plates. Possible explanations for this include: (1) transcription of related flagella biosynthesis genes was restricted and led to the loss of flagella and defective motility, such as in *Aeromonas hydrophila* [35]; (2) the levels of 3′, 5′-cyclic diguanylic acid (c-di-GMP) were altered by mutation of the *lsr* operon, such as seen in *Clostridium difficile*, culminating in reduced motility [37]. In our subsequent transcriptome assay, the decreased transcription of some flagella biosynthesis genes, such as *fliI*, *fliF*, *flhA*, *flhB*, *motB*, and *fliK*, supported this. However, the molecular mechanisms involved require further analysis.

Our previous studies showed that the *luxS* gene was associated with the pathogenesis of APEC, and that mutation of this gene attenuated the virulence of APEC [1]. However, the precise role of AI-2 signaling in APEC is not clear and remains to be demonstrated whether the *lsr* operon is involved in mediating the virulence of APEC. In this study, the LD_50_ of APEC94Δlsr(Cm) was decreased by 294-fold. A similar trend was also observed for bacterial survival in an in vivo assay. Distinct attenuation of the bacterial load in the blood, liver, spleen, and kidneys of ducks infected with APEC94Δlsr(Cm) was evidenced compared with ducks infected with wild-type strain APEC94. The results of animal experiments indicated that the Lsr system is involved in mediating the virulence of APEC but the mechanism responsible for the decreased virulence of APEC94Δlsr(Cm) remains to be determined.

This phenomenon might be caused by down-regulation of certain virulence genes including those involved in mediating bacterial motility, iron metabolism, and survival in host cells. The motility assay showed that the motility of APEC94Δlsr(Cm) was impaired, confirming the role of the *lsr* operon. This was further supported by transcriptomic analysis of the APEC94 and APEC94Δlsr(Cm) strains. The expression of other genes was also affected by deletion of the *lsr* operon, leading to significantly decreased transcription levels. For example, the *fepG* and *fepE* genes that encode ferric proteins involved in enterobactin uptake and export were downregulated in expression by 2.8368- and 4.2153-fold, respectively [38, 39]. In many *E. coli* and *S. typhimurium* strains, the ferric enterobactin transporter facilitates iron uptake and plays a role in the defense against various sources of oxidative stress [39–41]. Another example was the *fimD* gene that encodes a fimbrial biogenesis outer membrane usher protein that is crucial for usher gating of type I pili, which was downregulated in expression by 16-fold [42]. Outer membrane proteins play an essential role in the rapid adaptation of Gram-negative bacteria to changes in their environment. Fimbriae, an important component of the outer membrane, plays a vital role in APEC during the adherence to and invasion of host cells and FimD is associated with virulence [43].

Furthermore, another 6 genes involved in type III export system, c-di-GMP and biofilm formation, were selected for further evaluating the effects of *lsr* operon on biological characteristics of APEC by q-PCR. For example, the flagellin protein FliD plays a role in flagellin polymerization. Deletion of *fliD* of *Salmonella* could induce a lower expression of immune genes in infected chicken embryo fibroblasts [28, 44]. FlhA is a membrane-protein subunit of the bacterial flagellar type III export system, which functions in many gram-negative pathogens to export virulence factors into host cells [29]. DosP was reported with multiple biological functions, such as cleaving both c-di-GMP and cAMP [30]. PgaA is required for the formation of polymeric β-1,6-*N*-acetyl-d-glucosamine (poly-β-1,6-GlcNAc), which has been implicated as an *E. coli* biofilm adhesin [31]. The FhuD is a necessary component of the hydroxamate siderophore transport system [32]. The present results showed that deletion of *lsr* decreased transcriptional levels of *fliD*, *flhA*, *dosP*, *pgaA* and *fhuD*, which also explained the possible reason for decreasing pathogenicity of *lsr* mutant strain. However, the transcriptional level of genes *pgaA* and *fhuD* did not restored in *lsr*-APEC94Δlsr. A possible reason was that the transcriptional level of *lsr* operon genes of complement strain were higher than that of wild strain, which affected the expression of *pgaA* and *fhuD* in *lsr*-APEC94Δlsr. The underlying mechanisms still need to be further studied.

In conclusion, our study showed that the distribution of the *lsr* operon was associated with different serotypes of APEC and affected the motility of bacterial cells, AI-2 uptake, and pathogenesis. This study provides a basis for further functional research into the role of *lsr* operon in APEC.

## Supplementary information

**Additional file 1. The distribution of** ***lsr*** **operon in APEC strains.**

**Additional file 2. Nucleotide sequence identities of APEC94 compared with** ***E. coli*** **MG1655 and** ***Salmonella*** **TL2.**

**Additional file 3. Amino acid sequence identities of APEC94 compared with** ***E. coli*** **MG1655 and** ***Salmonella*** **TL2.**
