# Supplementary material for: Lsr operon is associated with AI-2 transfer and pathogenicity in avian pathogenic Escherichia coli
Source: Vet Res. 2019 Dec 12;50:109. doi: 10.1186/s13567-019-0725-0 (PMC6909531; doi:10.1186/s13567-019-0725-0)
Supplement: Supplementary file 1 — Additional file 1. The distribution of lsr operon in APEC strains. [file 13567_2019_725_MOESM1_ESM.doc]

**Additional file 1** The distribution of lsr operon in APEC strains

| strain | serotype | *lsrK* | *lsrR* | *lsrA* | *lsrC* | *lsrD* | *lsrB* | *lsrF* | *lsrG* |
| --- | --- | --- | --- | --- | --- | --- | --- | --- | --- |
| EG1407 | O_1_ | + | + | + | + | + | + | + | + |
| CE46 | O_1_ | － | － | － | － | － | － | － | － |
| C2 403 | O_1_ | － | － | － | － | － | － | － | － |
| C2402 | O_1_ | － | － | － | － | － | － | － | － |
| APECO1 | O_1_ | － | － | － | － | － | － | － | － |
| CE48 | O_1_ | + | － | － | － | + | － | － | － |
| CE47 | O_1_ | － | － | － | － | － | － | － | － |
| AH46 | O_1_ | + | + | + | + | + | + | + | + |
| AH48 | O_1_ | + | + | + | + | + | + | + | + |
| AH51 | O_1_ | + | + | + | + | + | + | + | + |
| FJ1-A | O_1_ | + | + | + | + | + | + | + | + |
| FJ1-11 | O_1_ | + | + | + | + | + | + | + | + |
| FJ1-29 | O_1_ | + | + | + | + | + | + | + | + |
| AH13 | O_1_ | + | + | + | + | + | + | + | + |
| AH15 | O_1_ | + | + | + | + | + | + | + | + |
| FJ1-20 | O_1_ | + | + | + | + | + | + | + | + |
| FJ1-2 | O_2_ | + | + | + | + | + | + | + | + |
| FJ1-3 | O_2_ | + | + | + | + | + | + | + | + |
| FJ1-9 | O_2_ | + | + | + | + | + | + | + | + |
| FJ1-14 | O_2_ | + | + | + | + | + | + | + | + |
| FJ1-15 | O_2_ | + | + | + | + | + | + | + | + |
| FJ1-23 | O_2_ | + | + | + | + | + | + | + | + |
| FJ1-24 | O_2_ | + | + | + | + | + | + | + | + |
| AH16 | O_2_ | + | － | + | + | + | + | + | + |
| AH26 | O_2_ | － | － | － | － | － | － | － | － |
| AH30 | O_2_ | － | － | － | － | － | － | － | － |
| DE17 | O_2_ | － | － | － | － | － | － | － | － |
| 5155 | O_2_ | － | － | － | － | － | － | － | － |
| XM | O_2_ | － | － | － | － | － | － | － | － |
| DE142 | O_2_ | － | － | － | － | － | － | － | － |
| DE164 | O_2_ | － | － | － | － | － | － | － | － |
| DE235 | O_2_ | － | － | － | － | － | － | － | － |
| DE205 | O_2_ | － | － | － | － | － | － | － | － |
| Dong O1 | O_2_ | － | － | － | － | － | － | － | － |
| Dong O2 | O_2_ | － | － | － | － | － | － | － | － |
| CE37 | O_2_ | － | － | － | － | － | － | － | － |

| strain | serotype | *lsrK* | *lsrR* | *lsrA* | *lsrC* | *lsrD* | *lsrB* | *lsrF* | *lsrG* |
| --- | --- | --- | --- | --- | --- | --- | --- | --- | --- |
| CE35 | O_2_ | + | － | － | － | + | － | － | － |
| CE38 | O_2_ | － | － | － | － | － | － | － | － |
| AH39 | O_78_ | － | － | － | － | － | － | － | － |
| AH50 | O_78_ | + | － | － | + | + | + | + | + |
| AH53 | O_78_ | + | + | + | + | + | + | + | + |
| AH54 | O_78_ | + | + | + | + | + | + | + | + |
| AH55 | O_78_ | + | + | + | + | + | + | + | + |
| AH61 | O_78_ | + | + | + | + | + | + | + | + |
| AH62 | O_78_ | + | + | + | + | + | + | + | + |
| AH63 | O_78_ | + | + | + | + | + | + | + | + |
| AH65 | O_78_ | + | + | + | + | + | + | + | + |
| AH70 | O_78_ | + | + | + | + | + | + | + | + |
| AH76 | O_78_ | + | + | + | + | + | + | + | + |
| FJ2-5 | O_78_ | + | + | + | + | + | + | + | + |
| DE01 | O_78_ | + | + | + | + | + | + | + | + |
| APEC94 | O_78_ | + | + | + | + | + | + | + | + |
| E937 | O_78_ | + | + | + | + | + | + | + | + |
| E1103 | O_78_ | + | + | + | + | + | + | + | + |
| DE05B | O_78_ | + | + | + | + | + | + | + | + |
| DE099 | O_78_ | + | + | + | + | + | + | + | + |
| DE102 | O_78_ | + | + | + | + | + | + | + | + |
| DE001B | O_78_ | + | + | + | + | + | + | + | + |
| E1102 | O_78_ | + | + | + | + | + | + | + | + |
| GE02 | O_78_ | + | + | － | + | + | + | + | + |
