# Supplementary material for: Lsr operon is associated with AI-2 transfer and pathogenicity in avian pathogenic Escherichia coli
Source: Vet Res. 2019 Dec 12;50:109. doi: 10.1186/s13567-019-0725-0 (PMC6909531; doi:10.1186/s13567-019-0725-0)
Supplement: Supplementary file 2 — Additional file 2. Nucleotide sequence identities of APEC94 compared with E. coli MG1655 and Salmonella TL2. [file 13567_2019_725_MOESM2_ESM.doc]

**Additional file 2 Nucleotide sequence identities of APEC94 compared with *E. coli* MG1655 and *Salmonella*** TL2

| APEC94 | *lsrK* | *lsrR* | *lsrA* | *lsrC* | *lsrD* | *lsrB* | *lsrF* | *lsrG* | *Lsr* |
| --- | --- | --- | --- | --- | --- | --- | --- | --- | --- |
| MG1655 | 1588/1603  (99%) | 940/954  (99%) | 1520/1536  (99%) | 1010/1029  (98%) | 985/993  (99%) | 1016/1023  (99%) | 867/876  (99%) | 288/291  (99%) | 8589/8673  (99%) |
| *Salmonella* TL2 | 1186/1598  (74%) | 684/969  (71%) | 1082/1549  (70%) | 765/1049  (73%) | 712/1009  (71%) | 786/1030  (76%) | 696/882  (79%) | 222/336  (66%) | 6357/8832  (72%) |
