# Supplementary material for: Lsr operon is associated with AI-2 transfer and pathogenicity in avian pathogenic Escherichia coli
Source: Vet Res. 2019 Dec 12;50:109. doi: 10.1186/s13567-019-0725-0 (PMC6909531; doi:10.1186/s13567-019-0725-0)
Supplement: Supplementary file 3 — Additional file 3. Amino acid sequence identities of APEC94 compared with E. coli MG1655 and Salmonella TL2. [file 13567_2019_725_MOESM3_ESM.doc]

**Additional file 3 Amino acid sequence identities of APEC94 compared with *E. coli* MG1655 and *Salmonella*** TL2

| APEC94 | *lsrK* | *lsrR* | *lsrA* | *lsrC* | *lsrD* | *lsrB* | *lsrF* | *lsrG* |
| --- | --- | --- | --- | --- | --- | --- | --- | --- |
| MG1655 | 528/530(99%) | 315/317(99%) | 506/511(99%) | 339/342(99%) | 330/330(100%) | 338/340(99%) | 287/291(99%) | 96/96(100%) |
| *Salmonella* TL2 | 438/530(83%) | 246/320(77%) | 383/511(75%) | 289/347(83%) | 272/333(82%) | 289/340(85%) | 259/291(89%) | 77/109(71%) |
